# Entometabolomics: applications of modern analytical techniques to insect studies

**DOI:** 10.1111/eea.12281

**Published:** 2015-02-14

**Authors:** Charles J.P. Snart, Ian C.W. Hardy, David A. Barrett

**Affiliations:** ^1^ Centre for Analytical BioscienceSchool of PharmacyUniversity of NottinghamUniversity Park CampusNottinghamNG7 2RDUK; ^2^ School of BiosciencesUniversity of NottinghamSutton Bonington Campus, LoughboroughLeicestershireLE12 5RDUK

**Keywords:** nuclear magnetic resonance, gas chromatography‐mass spectrometry, liquid chromatography‐mass spectrometry, multivariate data analysis

## Abstract

Metabolomic analyses can reveal associations between an organism's metabolome and further aspects of its phenotypic state, an attractive prospect for many life‐sciences researchers. The metabolomic approach has been employed in some, but not many, insect study systems, starting in 1990 with the evaluation of the metabolic effects of parasitism on moth larvae. Metabolomics has now been applied to a variety of aspects of insect biology, including behaviour, infection, temperature stress responses, CO_2_ sedation, and bacteria–insect symbiosis. From a technical and reporting standpoint, these studies have adopted a range of approaches utilising established experimental methodologies. Here, we review current literature and evaluate the metabolomic approaches typically utilised by entomologists. We suggest that improvements can be made in several areas, including sampling procedures, the reduction in sampling and equipment variation, the use of sample extracts, statistical analyses, confirmation, and metabolite identification. Overall, it is clear that metabolomics can identify correlations between phenotypic states and underlying cellular metabolism that previous, more targeted, approaches are incapable of measuring. The unique combination of untargeted global analyses with high‐resolution quantitative analyses results in a tool with great potential for future entomological investigations.

## Introduction

The development of metabolomic methodologies is ongoing and has been applied to an expanding range of fields. Within the field of entomology, metabolomic techniques have been used to reveal biochemical information to aid in the understanding of physiology and behaviour. While there is an increasing amount of data available regarding gene regulation (Harshman & James, 1998; Imler & Bulet,^2005^; Smith et al., 2008), transcriptomics (Pauchet et al., 2009; Mittapalli et al., 2010; Zhang et al., 2010) and proteomics within insect models (Stadler & Hales, 2002; Wolschin & Amdam, 2007; Cilia et al., 2011), information on the role of differential metabolic states in regulating insect behaviours and phenotypes has remained comparatively scarce. Those investigations that have been conducted have indicated that the ‘‐omics’ approach is increasingly promising for entomological applications (Lenz et al., 2001; Coquin et al., 2008; Kamleh et al., 2008; Aliferis et al., 2012).

Metabolomics is one of the newest ‘‐omics’ technologies, and has rapidly expanded over the last decade, providing an integral new approach to the study of biological systems (Dettmer & Hammock, 2004; Rochfort, 2005). Although this field was first defined by Oliver et al. (1998) as ‘the quantitative measurement of the dynamic multi‐parametric metabolic response of living systems to pathophysiological stimuli or genetic modification’, some entomological investigations employing a recognisably metabolomic approach pre‐date the adoption of the term (e.g., Thompson et al., 1990). The growth of metabolomics is associated with the recent incorporation of high‐throughput methodologies, a coupling of classical analytical methodologies with automated processing technologies. This approach aims to generate as complete a metabolite profile within a given system as possible, and catalogue any metabolic fluctuations generated by a particular environmental condition or perturbation, with metabolites defined as molecules that are necessary for, or involved in, a particular metabolic process. The discipline has generated new insights into the subtle metabolic perturbations that exist within toxicology (Robertson et al., 2011), drug functionality (Kell, 2006), disease states (Schnackenberg, 2007), ageing (Schnackenberg et al., 2007), and overall cellular function (Nielsen, 2003).

Metabolomics possesses some advantages over the more established ‘‐omics’ approaches of genomics, transcriptomics, and proteomics. In particular its focus on ‘downstream’ cellular functions allows conclusions to be drawn regarding the functional metabolic phenotype of an organism. This form of analysis requires no prior knowledge of the genome of the organism under study, allowing useful biochemical data to be gathered even in the absence of full characterisation. Furthermore, the metabolomic approach provides a snapshot of the functional metabolic phenotype by detecting the full metabolome of a tissue or organism under a particular physiological state. Combined with the use of high‐throughput analytical techniques, such as nuclear magnetic resonance (NMR) and mass spectrometry (MS) (Reo, 2002; Dettmer et al., 2007), and the development of modern pattern recognition and multivariate data analysis software, metabolomics has become an effective way of summarising large changes in cell phenotype in terms of the fluctuations of a small number of metabolic pathways.

There are already some excellent reviews of current uses of metabolomic technologies, along with an extensive background to the field and its history (Nicholson & Wilson, 2003; Rochfort, 2005; Lindon & Nicholson, 2008; Heather et al., 2013), and several have focused on the use of metabolomics in particular fields of study, such as ecology (ecometabolomics) (Bundy et al., 2009; Jones et al., 2013; Lankadurai et al., 2013). In this review, we focus on metabolomics studies as they have been, and can be, applied to insect study systems. Although the number of insect studies employing a metabolomic approach has increased over the last decade, the total number of publications remains low (<50). Existing publications vary widely in their utilisation of data acquisition and analysis approaches, along with their reporting of technical parameters. Many of these parameters are required for independent assessments of the veracity of a particular study's findings; lack of their reporting can cast doubt on aspects such as insect rearing and collection, instrument stability, and data analysis. We aim to generate recommendations for improving this disparity by critically reviewing existing studies. As no prior review has attempted to critique purely ‘entometabolomic’ studies we also briefly summarise some of the more novel applications of this methodology and provide a catalogue of current literature (Table 1).

**Table 1:** Insect metabolomics studies

Insect order	Species	Research topic	Sample type	Techniques utilised	Conclusions
Diptera	*Aedes aegyti*	Juvenile hormone regulation	Solvent extract	HPLC‐FD*	Mevalonate and juvenile hormone pathways are highly dynamic and linked to reproductive physiology^1^
	*Belgica antarctica*	Temperature stress response	Solvent extract	GC‐MS	Freezing and desiccation are associated with increases in metabolites associated with carbohydrate metabolism and a decrease in free amino acids^2^. Shifts in metabolite pools are associated with changes in gene regulation related to dehydration^3^
	*Chymomyza costata*	Cryopreservation	Solvent extract, biofluid	GC‐MS, LC‐MS	Survival of cryopreservation is associated with increased proline levels in larval tissues^4^
	*Drosophila melanogaster*	Metabolomic profiling	Solvent extract, biofluid	GC‐MS, LC‐MS	Cold shock disturbs short‐ and long‐term cellular homeostasis^5^^,^ 6 ^,^ 7 ^,^ 8. Inbreeding, both in the absence and the presence of temperature stress, alters metabolic processes^9^. Lower rates of glycolysis occur in adapted flies undergoing hypoxia^10^^,^ 11 ^,^ 12. Age‐related decline of hypoxia tolerance is linked to reduced recovery of mitochondrial respiration^13^. >230 metabolites profiled across four *Drosophila* subspecies^14^^,^ 15. Bowman‐Birk inhibitor disrupts energy metabolism^16^. Long‐term cold acclimation modifies the larval metabolome^12^. Absolute quantification of 28 phospholipids^17^. Larvae with the y mutation have altered lysine metabolism^18^. CO_2_ exposure causes metabolic changes during short term recovery^19^. Infection by *Listeria monocytogenes* results in loss of energy store regulation^20^. Developmental and adult cold acclimation strongly promoted cold tolerance and restored metabolic homeostasis^21^
		Temperature stress responses			
		CO₂ anaesthesia			
		Bacterial infection			
		Hypoxia			
	*Drosophila montana*	Temperature stress responses	Solvent extract	GC‐MS, LC‐MS	Seasonal variations in thermoperiod are correlated with differential expression of myo‐inositol, proline and trehalose^22^
	*Sarcophaga crassipalpis*	Temperature stress response	Solvent extract	GC‐MS, 1D NMR	Rapid cold‐hardening elevates glycolysis associated metabolites whilst reducing levels of aerobic metabolic intermediates^23^
Hemiptera	Aphids (multiple species)	Trehalose analysis	Solvent extract, biofluid	1D NMR	High concentrations of trehalose are present in aphid hemolymph^24^. Removal of bacterial–insect symbiosis reduced expression of dietary metabolites, including essential amino acids^25^
		Insect–bacterial symbiosis			
Hymenoptera	*Apis mellifera*	*Nosema ceranae* infection	Solvent extract, biofluid	GC‐MS, LC‐MS	Exposure to infectious pathogens and neonicotinoid pesticides results in altered larval and adult metabolism^26^^,^ 27
		Pesticide exposure			
	*Praon volucre*	Diapause induction	Solvent extract	GC‐MS	Cold acclimation eliminated cryo‐stress associated homeostatic perturbations^28^
	*Venturia canescens*	Temperature stress responses	Solvent extract	GC‐MS	Increases in cold tolerance are associated with the accumulation of cryoprotective metabolites^29^
Lepidoptera	*Helicoverpa armigera*	Diapause induction	Solvent extract	GC‐MS, MALDI‐TOF	Diapause induces metabolic alterations associated with photoperiodic information and energy storage^30^
	*Manduca sexta*	Host parasitism	Biofluid	1D NMR	Insect parasitism enhances glucogenesis induction and halts lipogenesis^31^^,^ 32. Concentrations of small molecule metabolites change alongside larval development^33^
	*Spodoptera frugiperda*	Metabolomic profiling	Solvent extract	LC‐MS	Identification of major pathways associated with cellular protein productivity^34^
	*Trichoplusia ni*	Metabolomic profiling	Solvent extract	LC‐MS	Major pathways associated with cellular protein productivity identified^34^
Orthoptera	*Chorthippus* (multiple species)	Metabolomic profiling	Solvent extract	GC‐MS	Determination of water soluble and lipid components of abdomial secretions of grasshoppers^35^^,^ 36
	*Locusta migratoria*	Developmental phase transition	Solvent extract	1D NMR	Onset of solitary‐group behavioural phase transitions are regulated by carnitine expression^37^
	*Schistocerca gregaria*	Social behaviour	Biofluid	1D NMR	Concentrations of trehalose and lipids were lower in the haemolymph of crowd‐reared than in solitary‐reared nymphs^38^
Phasmatodea	*Anisomorpha buprestoides*	Venom analysis	Biofluid	1D, 2D NMR	Stick insect defence secretions contain high levels of glucose, lysine, histodine, serotonin and sorbitol^39^
	*Peruphasma schultei*	Venom analysis	Biofluid	1D NMR	Individual insects produce different stereoisomeric mixtures^40^
Plecoptera	*Dinocras cephalotes*	Hypoxia	Solvent extract	1D NMR/DI‐MS	Metabolic shifts associated with heat stress are more pronounced under hypoxia^41^

*High‐Performance Liquid Chromatography with Fluorescence Detection.

^1^Rivera‐Perez et al. (2014); ^2^Michaud et al. (2008); ^3^Teets et al. (2012); ^4^Koštál et al. (2011b); ^5^Malmendal et al. (2006); ^6^Overgaard et al. (2007); ^7^Malendal et al. (2013); ^8^Williams et al. (2014); ^9^Pedersen et al. (2008); ^10^Feala et al. (2008); ^11^Feala et al. (2009); ^12^Koštál et al. (2011a); ^13^Coquin et al. (2008); ^14^Kamleh et al. (2008); ^15^Kamleh et al. (2009); ^16^Li et al. (2010); ^17^Hammad et al. (2011); ^18^Bratty et al. (2012); ^19^Colinet & Renault (2012); ^20^Chambers et al. (2012); ^21^Colinet et al. (2012a); ^22^Vesala et al. (2012); ^23^Michaud & Denlinger (2007); ^24^Moriwaki et al. (2003); ^25^Wang et al. (2010); ^26^Aliferis et al. (2012); ^27^Derecka et al. (2013); ^28^Colinet et al. (2012b); ^29^Foray et al. (2013); ^30^Zhang et al. (2012); ^31^Thompson et al., (1990); ^32^Thompson (2001); ^33^Phalaraksh et al. (2008); ^34^Monteiro et al. (2014); ^35^Buszewska‐Forajta et al. (2014b); ^36^Buszewska‐Forajta et al. (2014a); ^37^Wu et al. (2012); ^38^Lenz et al. (2001); ^39^Zhang et al. (2007); ^40^Dossey et al. (2006); ^41^Verberk et al. (2013).

The specific criteria for inclusion of an insect study into this review were based on whether an investigation was recognisably metabolomic in nature. As there is debate as to the exact definition of metabolomics (Oliver et al., 1998; Beecher, 2003; Ellis et al., 2007), we include studies according to the classification of recognised metabolomic approaches (including metabolomic fingerprinting and profiling) outlined by Goodacre et al. (2004). This review primarily focuses on the analytical techniques of NMR and MS, as these are the most commonly employed instruments within metabolomic studies. We begin by briefly outlining specific methodological aspects of how entometabolomic studies are carried out and then review the current range of studies employing this approach. As optimised protocols for ecometabolomics exist, the technical aspects of this review are limited to the discussion of sampling issues specific to entomological investigations. We conclude by critiquing the current state of the field and offering recommendations for future investigations.

## Establishing a metabolomic workflow

**Figure 1:**
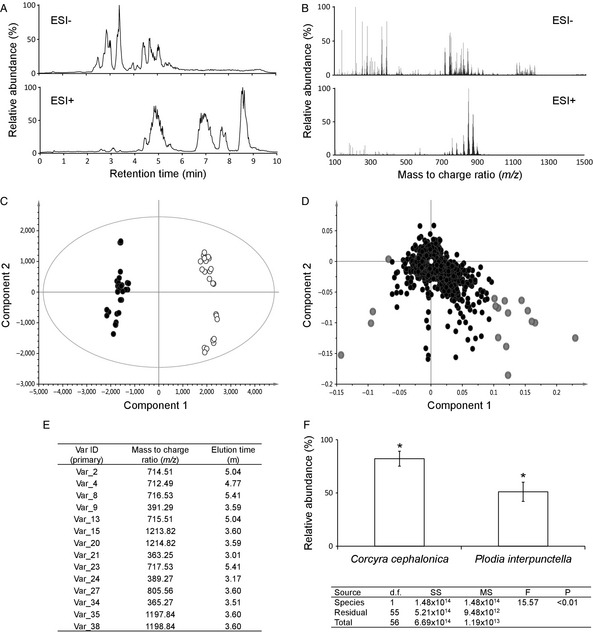
A sample data processing workflow. This investigation assessed differences in the larval metabolome across two pyralid moth species: rice moth (*Corcyra cephalonica* Stainton) and Indian mealmoth (*Plodia interpunctella* Hübner) (C Snart, unpubl.). Lipid extracts were generated using a modified methanol‐chloroform‐water extraction protocol and analysed using LC‐MS (A and B). LC‐MS chromatograms were aligned to a common reference sample and framed using the Thermo SIEVE (Thermo Fisher Scientific, Waltham, MA, USA) processing software. Aligned and framed data were then exported to the statistical software SIMCA 13.0.3 (Umetrics, Umeå, Sweden) and analysed using principle component analysis (PCA) (C and D). Group clustering of samples based on the two experimental groups was confirmed in the negative electrospray ionisation (ESI) mode PCA analysis (C). The two treatment groups were defined and an PLS‐DA analysis was utilised to directly compare between the two groups (R2X = 0.706, R2Y = 0.988, Q2 = 0.98). A loadings plot was utilised to aid in identifying major differences between the two groups (D). Group‐to‐group comparisons were used to highlight loadings (highlighted in grey) associated with the two groups. These differential loadings were examined for their associated mass‐to‐charge ratios (*m*/*z*) and elution times (E). Using these values, variable ID 9 was identified as a cholesterol derivative based on consultation with online metabolite databases [LIPID MAPS and the Human Metabolome Database (HMDB)]. Further qualitative data for this metabolite were generated using the Thermo XCALIBUR software (Thermo Fisher Scientific). Mean relative abundances (± 1 SD) are shown on a bar chart (F) and ANOVA found a significant difference in metabolite level between the two groups (F).

A large number of reviews and methodological publications already exist outlining the major analytical and statistical steps involved in the establishment of an appropriate workflow for conducting a metabolomics, or indeed any ‘‐omics’, investigation (e.g., Fiehn, 2002; Broadhurst & Kell, 2006; Tiziani et al., 2011; Ibáñez et al., 2013; Nikolskiy et al., 2013). Furthermore, there are comprehensive reviews of ecometabolomics (Sardans et al., 2011; Rivas‐Ubach et al., 2013), as well as methodological protocols optimised for insect tissues (Zhang et al., 2007; Kamleh et al., 2008). Due to the existence of these publications, we limit our discussion of the technical aspects of metabolomics to a brief consideration of specific sample collection problems entometabolomic studies may particularly encounter. We also supply a simplified workflow of a model entometabolomic study (Figure 1).

### Sample preparation

Despite the existence of standardised sample preparation methodologies (Folch et al., 1957; Hara & Radin, 1978; Wu et al., 2008), problems may arise with specific organisms, in the case of insect studies this is often specifically related to low biomass (Lorenz et al., 2011; Marcinowska et al., 2011; Kim et al., 2013). As the majority of extraction methodologies are tailored for larger biomass samples, the volumes and ratios associated with these approaches may require adaptation if adopted for low biomass investigations, such as in Wu et al. (2008). Furthermore, in order to prevent cross‐contamination, it may be optimal to clean the organism using high‐purity water, or another appropriate solvent, prior to snap freezing. This is particularly important for entomological investigations, due to many laboratory insect populations being reared in groups where the surface of the specimen may be exposed to culture media and/or faecal matter that could affect the outcome of analysis if detected. Several common culturing components, including honey, glycerol, and ethanol, are readily detectable in metabolomic analysis, particularly in the case of ^1^H NMR spectroscopy (Phalaraksh et al., 2008). Diet should also be considered, particularly as highly sensitive analytical approaches may also detect differences in gut composition. A possible approach to eliminating this issue would be to perform similar extractions and analytical profiling of the insect diet: dietary spectral data could then be compared with experimental samples, and used to rule out any observed background resonances or ions.

## Current approaches to entometabolomics

Although many applications are currently only represented by relatively few studies, entometabolomics has contributed to the understanding of such topics as hypoxia (Coquin et al., 2008; Feala et al., 2008), insect–bacterial symbiosis (Wang et al., 2010), behavioural ecology (Lenz et al., 2001), parasitism (Thompson et al., 1990), development (Phalaraksh et al., 2008; Wu et al., 2012), infectious diseases (Aliferis et al., 2012), the effects of commercial pesticides (Derecka et al., 2013), and temperature‐dependent stresses (Michaud & Denlinger, 2007; Michaud et al., 2008; Koštál et al., 2011a,b) (Table 1). Simultaneously, metabolomic investigations have indirectly generated information about insect life histories; particularly work focusing on plant‐insect interactions (Hunt et al., 2006, 2010; Faria et al., 2007; Gattolin et al., 2008; Jansen et al., 2009; Leiss et al., 2011). The adoption and output of these approaches has steadily increased throughout the last decade, with novel applications appearing almost annually.

Many recent investigations involving insect tissues fall within the loosely defined field of ecometabolomics (Michaud et al., 2008; Sardans et al., 2011). The majority of entometabolomic studies have focused on single factor approaches, often without taking into account that numerous factors can affect the metabolome (e.g., time since the animal last fed, its health status and its age, the effects of varying the circadian rhythm). The metabolome is in fact highly dynamic, and this repeated fluctuation makes it virtually impossible to characterise every metabolite present within an individual insect (Sardans et al., 2011). Further, to obtain estimates of the natural metabolomic state, it is often desirable to perform entomological studies in the field, even though it may not be possible to regulate certain behavioural or physiological factors (e.g., diet and feeding time, photoperiod). A transition between field conditions and final laboratory‐based metabolomic analysis can also result in metabolomic perturbations. Minimising potential sources of external biological variation is critical for a metabolomic experimental design, as a result it is particularly important to consider external sources of variation that may result from such a transition (e.g., maintaining change in wild diet to laboratory‐based diet, stress generated due to change in environment). Foray et al. (2013) deliberately attempted to avoid such variation by only allowing specimens to undergo short‐term acclimation prior to metabolomic analysis, whilst Derecka et al. (2013) avoided any such acclimation by conducting metabolome quenching in the field. However, field quenching relies on constancy of several factors, including the availability of a quenching mechanism, sampling point consistency, and maintenance of the sample at sub‐zero temperatures. In the case of laboratory studies involving laboratory cultures the existence of many established insect stocks can mitigate this, as the long‐term culturing of specimens in a stable environment may largely eliminate environmental perturbations.

### Metabolite profiling

The majority of MS‐based insect metabolome studies have utilised the model organism *Drosophila melanogaster* Meigen (Kamleh et al., 2008, 2009; Hammad et al., 2011; Koštál et al., 2011a,b; Bratty et al., 2012; Colinet & Renault, 2012). This is not surprising, given that the combination of a large well‐characterised stock of genetic mutants, genetic tractability, and a known organismal complexity make an ideal choice for generating insight into the composition and organisation of metabolic networks (Kamleh et al., 2008, 2009). The use of high‐resolution analytical techniques also provides a solution to a remaining disadvantage, that of low biomass.

By combining this approach with the use of pooled samples, >200 metabolites have been identified using liquid chromatography (LC)‐MS (Kamleh et al., 2008), including absolute lipid quantification (Kamleh et al., 2009) and validation (Hammad et al., 2011). These studies further indicated the practicality of LC‐MS to detect differences between extremely low‐biomass insect treatments, to the extent of being able to differentiate between individual *Drosophila* belonging to different subspecies or mutant types.

**Figure 2:**
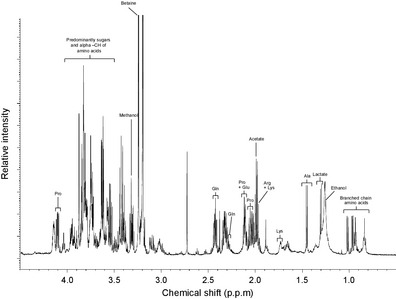
^1^H 600 Mz aliphatic NMR spectrum of the larvae of the rice moth, *Corcyra cephalonica*. Spectral information was generated through the use of modified Folch methanol‐water‐chloroform. Repeated investigations into the NMR profile of larval haemolymph have shown a remarkably conserved spectral structure (C Snart, ICW Hardy & DA Barrett, unpubl.). With the exception of the overlapping sugar‐amino acid spectral region situated at 4.0–3.4 p.p.m., a high proportion of commonly observed peaks is readily assignable through simple literature comparison (Phalaraksh et al., 2008). Although exact spectral positions can vary based on the particular operating frequency of the instrument and environmental fluctuations, existing characterisations of tissue/haemolymph NMR spectra can aid in the normally extensive process of individual peak identification. Ala = alanine, Arg = arginine, Gln = glutamine, Glu = glutamic acid, Lys = lysine, Pro = proline.

Many current NMR‐based analyses of the insect metabolome have focused on characterising the properties of insect biofluids, with specific focus on the composition of larval and pupal haemolymph (Thompson et al., 1990; Lenz et al., 2001; Thompson, 2001; Phalaraksh et al., 2008). These studies provided expanded information regarding the composition of amino acids, organic acids, sugars, and the role of ethanol. Perhaps the most important aspect of these early studies is the generation of an available list of common insect haemolymph metabolites (Phalaraksh et al., 2008); this is applicable for metabolite identification in both insect and crustacean investigations (Poynton et al., 2011). The list includes a large number of high‐concentration molecules, the variation of which has been related to social behaviour (Wu et al., 2012) and heat stress (Michaud & Denlinger, 2007). However, the detection of alterations of metabolites present at a low concentration can be problematic, due to the over representation of many sugars within the 4–3 p.p.m. region of most NMR spectra generated from both haemolymph and full tissue extractions (Figure 2). Any attempt to assign identifications to resonances within this region would require further spectral information, such as two‐dimensional (2D) NMR, an approach that has been utilised by more recent studies (Malmendal et al., 2006; Overgaard et al., 2007; Coquin et al., 2008; Hawes et al., 2008; Pedersen et al., 2008; Feala et al., 2009).

### Hypoxia and anaesthesia

The use of LC‐/gas chromatography (GC)‐MS and NMR has generated new insights into the metabolic effects of hypoxia in insect study systems, focusing on the regulation of glycolysis (Feala et al., 2008, 2009; Verberk et al., 2013) and fluctuations in the concentrations of free metabolites, such as proline, alanine, lactate, and acetate (Coquin et al., 2008; Koštál et al., 2011b). These studies also indicated that ageing was associated with a decline in hypoxia recovery; this recovery was linked to changes in free metabolite concentration after re‐oxygenation. These investigations illustrate another benefit of high‐resolution analytical techniques when experimental tissue volumes are low. The tissue of interest, the cardiac muscle, within individual flies was not present in significant quantities to be of use in biochemical investigations. This type of analysis would also face difficulty in consistently performing an appropriate dissection protocol on an insect of this size. The less direct route of metabolic modelling offered by NMR or MS allowed this limitation to be overcome.

Many entomological investigations require some form of anaesthesia to allow handling, colony maintenance, or identification (Vinuela, 1982; Nicolas & Sillans, 1989; Ashburner et al., 2005). Direct CO_2_ exposure is a widespread method of anaesthesia within entomological studies, despite a number of reported side effects concerning reproductive and behavioural traits which may impact physiological and metabolic traits (Nilson et al., 2006; Champion de Crespigny & Wedell, 2008). Colinet & Renault (2012) used GC‐MS to investigate the metabolic effects of this exposure, both in terms of an acute exposure, and a long‐term recovery, showing that CO_2_ exposure resulted in acute metabolic changes that are present for 14 h. These changes were directly related to the anoxic conditions related to cardiovascular disruption. However, there was no indication of long‐term alterations after a 24‐h period, allowing the conclusion that CO_2_ anaesthesia is an acceptable procedure when a longer recovery time is possible. With the exception of Chambers et al. (2012), the studies cited in this review either avoided the use of CO_2_ anaesthesia, or accounted for this recovery period in their methodology (Overgaard et al., 2007).

### Insect development and social behaviour

^1^H NMR spectroscopy has been used to compare the haemolymph metabolome of nymphs of the desert locust, *Schistocerca gregaria* (Forskal), reared under both solitary and gregarious conditions (Lenz et al., 2001). A number of metabolites varied across rearing conditions, including trehalose, lipids, acetate, and ethanol. However, later studies generated contradictory haemolymph metabolite identifications (Phalaraksh et al., 2008). A similar investi‐gation utilised MS to examine solitary‐gregarious behavioural transitions in a related locust species, *Locusta migratoria* (L.) (Wu et al., 2012). Direct comparisons of the haemolymph of solitary and gregarious phase locusts using high‐performance LC‐MS and GC‐MS identified 319 metabolites exhibiting differential concentrations between the two phenotypes. Of these, carnitine was identified as a key differential metabolite regulating locust phase transition from solitary to gregarious, alongside its acyl derivatives. This study presents the first example of an MS approach being applied to link differences in insect behaviour with the underlying metabolomic state.

### Temperature‐dependant stress responses

The most common topic of insect metabolomics concerns fluctuations in the metabolome when subjects are exposed to a range of extreme temperatures (Table 1) due to both seasonal and daily cycles (Malmendal et al., 2006; Pedersen et al., 2008). Many insect species have developed biochemical, behavioural, or physiological adaptations to minimise the potential damage from these fluctuations (Michaud & Denlinger, 2007). Extensive study of the insect metabolome (particularly of *Drosophila*) under different temperature stresses has confirmed that these perturbations can have both short‐ and long‐term effects on metabolite concentrations (Michaud et al., 2008), along with a more general effect on cellular homeostasis (Malmendal et al., 2006). These differences in metabolite fingerprint are conserved across various temperature treatments, and indicated that the concentrations of several major metabolites were significantly altered by heat‐shock, including, but not limited to, primary amino acids, ATP, acetate, and glycogen (Malmendal et al., 2006). Notably, these differences do seem to be largely conserved across a number of species (Moriwaki et al., 2003; Phalaraksh et al., 2008).

Various studies, utilising both NMR and MS, have noted a similar effect from cold‐shock, with particular focus being placed on the inducement of an elevated level of glycerol. Alongside this, increases in sorbitol, proline, alanine, glutamine, pyruvate, glucose, and urea, and parallel decreases in trehalose, mannose, beta‐alanine, and ornithine have been identified. Of these, the essential role of proline in surviving cold‐shock has been documented using GC‐MS/LC‐MS in a study involving the survival of the drosophilid fly, *Chymomyza costata* (Zetterstedt), when submerged in liquid nitrogen during diapause (Koštál et al., 2011b). Similar variations were noted in regards to seasonal variation in thermoperiod (Vesala et al., 2012), whilst contrasting thermal environments during insect development indicated differentiation in the levels of glucose, fructose, alanine, and glycine, along with an accumulation of polyamines. Potential alterations in metabolites associated with energy metabolism also suggested an alteration in energy metabolism, similar to that observed after cold acclimation in *Drosophila* (Koštál et al., 2011a; Colinet et al., 2012a). This may also confirm findings by Colinet et al. (2012b), who demonstrated similar disruptions in energy metabolism under diapause in the aphid parasitoid *Praon volucre* (Haliday).

### Insect–plant interactions

Some studies have indirectly used a metabolomic approach to draw conclusions about plant‐insect interactions (Allwood et al., 2008; Jansen et al., 2009; Leiss et al., 2011; Misra et al., 2010). One investigation focused on the effects of herbivore by different instars of the beet armyworm, *Spodoptera exigua* Hübner. Through a combination of 1D and 2D ^1^H NMR, Widarto et al. (2006) were able to differentiate significant alterations in the metabolome of *Brassica rapa* L. leaves after the initiation of feeding damage. Spectral investigation, conducted using principle component analysis (PCA), indicated an increase in the levels of glucose, ferulic acid, and gluconapin in response to feeding by second‐instar *S. exigua*, compared with an increase in alanine and sinapoyl malate for fourth‐instar feeding. By comparison, larvae of the moth *Plutella xylostella* L., a more specialist herbivore, elicited an increase in gluconapin, glucose, feruloyl malate, sinapoyl malate, and threonine. This study again demonstrated some of the advantages associated with two dimensional NMR, as it was able to reduce assignment problems associated with overlapping spectral traces.

### Integrated metabolomic approaches

An emerging trend within metabolomic research is to combine different high‐throughput technologies to generate an integrated ‘‐omics’ based approach. To date, four separate investigations have analysed insect tissues using a combination of metabolomics with either proteomics (Wang et al., 2010; Zhang et al., 2010) or transcriptomics (Teets et al., 2012; Derecka et al., 2013) and, to some extent, genomics (Derecka et al., 2013). These approaches attempted to correlate genomic/transcriptomic information with more ‘down‐stream’ metabolomic or proteomic datasets. An integrated study investigated the symbiotic bacterial system present in the pea aphid, *Acyrthosiphon pisum* (Harris) (Wang et al., 2010); along with the metabolomic aspect of the investigation, the aphid proteome was also subject to analysis. Utilising dietary antibiotics to eliminate the bacterium *Buchnera aphidicola* Munson et al., metabolomic analysis indicated alterations in metabolite and protein abundance (Wang et al., 2010). These changes were dominated by decreased essential amino acid abundance and an increase in non‐essential amino acids. These findings also indicated that the bacterial proteome/metabolome is more substantially affected by antibiotic treatment than by dietary manipulation. The metabolomic‐proteomic approach was similarly conducted by Zhang et al. (2012) to examine the brain of larval cotton bollworm, *Helicoverpa armigera* (Hübner), concurrent with artificial induction of seasonal diapause. This integrated approach clarified the control mechanisms that underlie the pre‐diapause phases, and showed that a wide range of metabolism‐related proteins and metabolites differ in concentration between diapause‐fated and non‐diapause‐fated larval brains.

A combined transcriptomic‐metabolomic approach (Teets et al., 2012) was used to investigate a different aspect of extremely cold environments, where water resources may be frozen for a large portion of the year, namely that of dehydration and desiccation tolerance. Using the Antarctic midge, *Belgica antarctica* Jacobs, an insect capable of surviving the loss of over 70% of body water, Teets et al. (2012) found that changes in gene expression associated with dehydration correlated strongly with changes in the metabolite pool. This study indicated that metabolic changes induced by the processes of desiccation and dehydration were remarkably similar, with changes occurring in such metabolites as glycolytic intermediates, lactate, proline, and citrate. These findings also indicate that metabolic responses are coordinated with changes in gene expression, a critical aspect of dehydration and desiccation responses. Transcriptomic comparisons with gene expression data derived from the arctic collembolan *Megaphorura arctica* (Tullberg), displayed little similarity in regulatory response (Teets et al., 2012), perhaps indicating that separate arthropod species have developed different compensatory mechanisms for low water availability (Teets et al., 2012).

## Entometabolomics: a critique of comparative studies

The ground‐breaking study by Thompson et al. (1990) on the metabolic effects of parasitism pre‐dates the formalisation of metabolomics as a field and also the adoption of current technical and statistical approaches that reduce the complexity of metabolomic datasets. Our critique therefore excludes this study. We also exclude studies that, although recognisably metabolomic in their approach (Goodacre et al., 2004), are focussed on reporting the profile of insects in a single species or state. We restrict our consideration to studies that primarily investigate the underlying metabolomic change that differentiates two or more phenotypic states. There have been 37 studies that meet this criterion, 33 of which were performed since the adoption of modern (i.e., post‐2006) reporting standards (Fiehn et al., 2006; Sumner et al., 2007). We suggest that improvements can be made in several areas, including sampling procedures, the countering of sampling and equipment variation, the use of sample extracts, statistical analyses, confirmation, and metabolite identification.

### Sampling procedures

As previously detailed, low biomass (<1 mg) has typically required modifications to established solvent extraction methodologies. A common approach for improving yield in current entometabolomic approaches has been to pool insect tissue samples (e.g., Kamleh et al., 2008, 2009; Koštál et al., 2011a,b); pooling also appears to have been used to make individual samples more representative of a given population (e.g., a honey bee colony, *Apis mellifera* L.; Aliferis et al., 2012). Whereas pooling has largely overcome the problem of low spectral complexity, there is a trade‐off with the number of replicates potentially available. Reduced replication, and hence lower statistical power during validation, increases the possibility of type II error (failing to reject an incorrect null hypothesis; Smith et al., 2011). Although low biomass may result in problems with yield, complex spectral information can be obtained from single large (>20 mg) insects (Lenz et al., 2001; Phalaraksh et al., 2008). Enhancement of the number of replicates within a treatment should typically take precedence when spectral yield is already substantial. Of the studies we consider, 63.6% (21/33) utilised adequate replication within each treatment (>5), as defined by current reporting standards (Fiehn et al., 2006; Goodacre et al., 2007; Sumner et al., 2007). Although sample sizes do not appear to have been adversely affected by pooling in these studies, it remains important to explicitly consider its desirability prior to experimental analysis.

### Tackling unwarranted variation

An organism's metabolome can be subject to fluctuations influenced by many environmental sources. Although these sources may seem minor, such as changes in diet or culture conditions, they can interfere with the composition of spectral and chromatographic data. As many insect cultures are maintained in a controlled environment, perturbations in temperature, photoperiod, and humidity from these sources are reduced. However, for a metabolomic approach to work correctly, it is also crucial to maintain uniformity across sample collection, extraction and processing conditions, along with analytical uniformity (Wishart, 2008). Sample extraction conditions in particular can have several sources of contamination and unnecessary variation. Perhaps the most vital step in initial sample collection, halting metabolomic activity, can be compromised by failing to maintain suitable experimental conditions during sample extraction and pre‐processing. Failure to maintain these conditions, usually by the use of ice‐cold solvents, can result in further alterations in the metabolome during extraction: this is undesirable as the metabolomic approach attempts to assess an organism's actual biochemical state. Unwanted variation can also occur if an extraction protocol requires a drying and reconstitution phase prior to analysis. For example, Li et al. (2010) utilised a rotary evaporator to dry samples at 43 °C for 3 h. Although high temperature may have affected the composition of the sample prior to analysis, it would also have advantageously decreased the time required for sample concentration. There can thus be a trade‐off between processing time and sample stability, in which case it may be preferable to use less disruptive drying protocols, such as N_2_ evaporation.

Imprecision of analytical equipment is another source of undesirable variation. The precision of an analytical approach is usually established through ‘technical replication’ which consists of separate analyses of sub‐samples of each experimental replicate. The standard deviations of the measurements for key metabolites can then be assessed to evaluate the stability of the analytical methodology. Of the 33 post‐reporting standards studies we are considering, only 15.2% (5/33) provided information regarding technical replication: all of these were able to adequately summarise this information in a single sentence within their materials and methods sections. Similarly, the stability of the chosen analytical method is often established through the continuous analysis of a pooled sample during a metabolomic experiment. Sub‐samples are expected to cluster centrally within any multivariate analysis and exhibit low variability throughout the analytical timeframe. Whilst this approach is commonly utilised during high‐sensitivity MS, some form of quality control sample can still be utilised by NMR spectroscopy. Despite this, only one investigation (Verberk et al., 2013) explicitly stated that a pooled quality control was used. The utilisation of a randomised sampling order, in order to reduce data skew stemming from instrument drift, was similarly reported by only a single study (Foray et al., 2013). It is possible that the remaining studies did not employ these common forms of validation. A more likely scenario is, however, that this detail was unreported due to its routine nature. Nevertheless, the lack of explicit reporting of quality control methods can cast doubt on the stability of a methodology, particularly in studies with large analytical timeframes. Another stability concern, specifically related to GC‐MS, is the automatisation of derivatisation prior to analysis; a process which ensures an identical processing time for each sample. Although studies utilising GC‐MS all provided information regarding derivatisation, only 18.7% (3/16) provided supporting information on this automation.

Photoperiod is one of the largest potential sources of variation in ecometabolomics (Beck, 1975; Koštál, 2006). Numerous studies have indicated that many metabolic pathways undergo photo‐period dependent shifts, including the amino acid (Fernstrom et al., 1979; Gattolin et al., 2008), carbohydrate (Das et al., 1993; Seay & Thummel, 2011), lipid (Turek et al., 2005; Seay & Thummel, 2011), nucleotide (Kafka et al., 1986; Fustin et al., 2012), and even xenobiotic pathways (Claudel et al., 2007). These shifts can be correlated with the onset and cessation of major behavioural processes, perhaps most notable for entometabolomics is the control of feeding behaviour (Seay & Thummel, 2011). Despite the critical role of the circadian clock in influencing physiological and behavioural rhythms, 33% (11/33) of post‐2006 studies provided no supporting information for the experimental photoperiod. Of the remaining studies, 59% (13/22) used methodology that explicitly attempted to avoid variations in sample collection time. However, this figure is influenced by a high proportion of diapause related publications (6/22) which often require photoperiod‐regulated induction prior to analysis. By comparison, 50% (2/4) of pre‐2006 studies provided supporting information for photoperiod and 25% (1/4) accounted for this potential variation during sample collection.

Although there is certainly room for improvement, it must be acknowledged that limiting the effects of photoperiod may not be practical for field‐based investigations that rely on very little laboratory acclimation to maintain accuracy (Foray et al., 2013). However, as previously stated, it may still be possible to harmonise sample quenching times for both individual samples and treatment groups (Derecka et al., 2013). Similarly, investigations focusing on changes in behavioural (e.g., solitary vs. gregarious) or physiological state (e.g., diapause, parasitized) rather than a specified treatment period may struggle to standardise sample collection time. A possible means of limiting variation may be to standardise time between the onset of the desired state and sample quenching.

### Effective use of sample extracts

There is a notable imbalance between metabolite classes examined in the studies we consider. Most (93.9%, 31/33) investigations have focused on polar metabolites, with a subset (21.2%, 7/33) attempting to also profile the non‐polar metabolites. Only one investigation (Derecka et al., 2013) focused exclusively on the non‐polar or lipid fragment generated by a methanol‐chloroform‐water based extraction. Buszewska‐Forajta et al. (2014a) primarily focused on analysing the lipid component, but these data were presented as the complement to polar data generated by Buszewska‐Forajta et al. (2014b), the two publications being derived from the same study.

The general lack of analysis of lipid fragments could be a major shortfall of entometabolomic investigations. This is due to the wide range of functions performed by the insect fat body, including energy storage and regulation (Arrese & Soulages, 2010), protein and nucleic acid production (Price, 1973), amino acid and carbohydrate production (Keeley, 1985), and metamorphosis (Mirth & Riddiford, 2007). Numerous entometabolomic publications have focused on polar metabolites that are involved in these same metabolic processes (e.g., Overgaard et al., 2007; Foray et al., 2013). Whilst it is possible that analysis of the non‐polar fragment did not yield differential results in these instances, lack of analysis of non‐polar fragments could lead to the loss of complimentary information concerning polar metabolite concentrations. As detailed above, complimentary data on polar and non‐polar metabolites can be generated by biphasic solvent extraction (Wu et al., 2008). With the recent emergence of a new sub‐division of ‘‐omics’ research, known as lipidomics (Wenk, 2005), it may prove advantageous to modify current solvent extraction protocols to favour a more lipocentric approach.

### Statistical analysis

As multivariate data analysis identifies potential biomarkers and underlying sources of variation, it has been widely utilised in entometabolomic studies to demonstrate appropriate separation between experimental classes. It is particularly important that any fitted statistical model is capable of describing a high degree of variation whilst possessing the capability of accurate prediction. As a result, attempts at providing a more defined set of reporting standards have emphasised the documentation of specific model parameters, particularly the ‘goodness’ of fit (termed R2X) and the ‘goodness’ of prediction (termed Q2X) (Eriksson et al., 2005). Within the studies we consider, only 11 of 33 reported an appropriate statistical model to establish separation between experimental treatments. A further four of these studies only provided rudimentary outline of the chosen statistical approach, without reporting relevant model parameters. Only 18.1% of the post‐2006 publications provided supporting information for separation [i.e., R2X or Q2 values for partial least squares‐discriminant analysis (PLS‐DA)], slightly fewer than in the pre‐2006 literature (1/4, 25%).

Although the establishment of overall separation and the provision of assessment parameters are important with highly multidimensional metabolomic data, further validation is also required to establish significant differences in individual metabolites between classes. Of the previous 33 publications, 26 utilised either parametric or non‐parametric statistical validation (78.8%). This is largely unchanged from pre‐2006 studies (3/4, 75%). Entometabolomic studies have encountered common problems with parametric testing; perhaps the most important of these is the potential for artificial inflation of significance through multiple‐hypothesis testing. Although statistical correction methods to control false discovery rates have long been available (Benjamini & Hochberg, 1995; Quinn & Keough, 2002; Verhoeven et al., 2005), they were only employed by one of the five pre‐2006 publications. The percentage is higher among post‐2006 studies (12/33, 36.4%). One particular recent investigation (Monteiro et al., 2014) did not apply any form of statistical testing, instead using an arbitrary cut‐off to conclude whether differences were present or absent. Although it is possible that this approach could accord with results of formal hypothesis testing, this is by no means certain, especially with low numbers of replicates (Quinn & Keough, 2002).

### Metabolite confirmation, identification, and function

It should also be noted that although both univariate and multivariate data analyses potentially generate lists of many differential compounds, it is important to confirm that this differentiation is based on real peaks, rather than noise. Furthermore, it is important to recognise that it is often not only difficult to identify the full range of spectral or chromatographic peaks, but also to correlate specific changes in the global metabolome with particular physiological or behavioural states. Data from both NMR and MS contain large overlapping peaks that can mask more subtle changes that may actually govern organism condition. Identification of what is accounting for these changes can be greatly enhanced by the application of knowledge of the biochemistry of the species concerned or of insects in general. Metabolite identification should employ as targeted an approach as possible, with earlier global profiling work acting as a screening process to identify potential metabolites of interest.

The importance of accurate identification is an enduring concern in metabolomic research, resulting in entometabolomic approaches utilising confirmation methodologies even in studies prior to the 2006 adoption of reporting standards. Current reporting for metabolomic research has become highly detailed, particularly with regard to the confirmation and validation of experimental findings. As accurate identification of differential biomarkers is a vital aspect of metabolomic investigations, many studies have employed further analytical methodologies, including tandem MS‐MS and 2D NMR. A number of investigations (45.5%) utilised and documented a further confirmation step, such as LC‐MS‐MS or 2D NMR, to identify metabolites more confidently. Including putative approaches, all but two publications (Li et al., 2010; Vesala et al., 2012) provided identification utilising comparisons with known spectral and chromatographic standards, spectral databases, or tandem MS‐MS fragmentation patterns. Whilst it is possible that the remaining two studies followed a similar identification protocol, this was not reported.

One area that exhibited consistent reporting was that of metabolite function. Although a metabolomic approach is capable of generating differential information about separate organism phenotypes, the generation of robust conclusions from these data requires a thorough understanding and examination of known metabolite pathways and information. Post‐2006 studies all included discussion of the potential function of differential metabolites, many correlating shifts within major pathways (e.g., glycolysis) and framing them within the context of major environmental perturbations (e.g., hypoxia) (Coquin et al., 2008). Pre‐2006 studies were similarly inclusive (4/4), although somewhat brief in the case of Lenz et al. (2001).

## Conclusions and recommendations

Metabolomics has been applied to a number of insect study systems in an effective manner, generating new insights into the mechanisms underlying aspects of biology including behaviour (Lenz et al., 2001), infection (Chambers et al., 2012), temperature stress responses (Malmendal et al., 2006; Li et al., 2010; Colinet et al., 2012a), CO_2_ sedation (Colinet & Renault, 2012), and bacteria–insect symbiosis (Wang et al., 2010). We are sure that this list of topics will expand in the near future.

Despite this success, there are opportunities to improve standards in terms of sample preparation, analytical methodology, statistical analysis, and reporting. The success or failure of a metabolomic investigation can depend on the rigour of the planning and developmental process preceding the investigation. To develop and employ an appropriate metabolomic workflow for an entomological study system, we recommend that future entometabolomic investigations follow points 1, 2, and 3 prior to experimental analysis, and points 4 and 5 before data analysis:

1. Plan the experiment carefully in advance with full consultation between the biological and analytical collaborators.
2. Minimise possible sources of environmental contamination or variation. Although the degree of elimination can be somewhat subjective depending on the goals of the investigation, this has been widely achieved through maintenance of a sterile, sub‐zero °C environment throughout sample quenching, extraction, storage, and, if possible, analysis.
3. Validate analytical methods. In order for an analytical method to be valid, it must be possible to demonstrate stability throughout the experimental timeframe. Recommended validation approaches include technical replication, randomisation of the sample injection order, and the use of a fixed internal standard.
4. Validate univariate and multivariate data analysis. Multivariate cross‐validation should be employed to demonstrate the closeness of fit for a discriminant analysis, whereas appropriate test statistics (and when relevant, descriptive statistics) should be provided for any univariate validation.
5. Provide robust supporting information for metabolomic data. Appropriate methodological and analytical metadata should be made available in order to maximise the utility of generated data to other researchers.

Overall, it is clear that the employment of a metabolomics approach can identify correlations between a phenotypic state and the underlying cellular metabolism that older, more targeted, approaches are incapable of measuring. This unique combination of untargeted global analysis with high‐resolution quantitative analysis presents an attractive tool for future entomological investigations.
